# Arms race in a cell: genomic, transcriptomic, and proteomic insights into intracellular phage–bacteria interplay in deep-sea snail holobionts

**DOI:** 10.1186/s40168-021-01099-6

**Published:** 2021-09-03

**Authors:** Kun Zhou, Ying Xu, Rui Zhang, Pei-Yuan Qian

**Affiliations:** 1 Department of Ocean Science and Hong Kong Branch of the Southern Marine Science and Engineering Guangdong Laboratory (Guangzhou), Hong Kong University of Science and Technology, Hong Kong, China; 2 Shenzhen University-HKUST Joint Marine Science Ph.D. Program, Shenzhen University, Shenzhen, 518060 China; 3 Shenzhen Key Laboratory of Marine Bioresource and Eco-environmental Science, College of Life Sciences and Oceanography, Shenzhen University, Shenzhen, 518060 China; 4 State Key Laboratory of Marine Environmental Science, College of Ocean and Earth Sciences, Xiamen University (Xiang’an), Xiamen, Fujian China; 5 Southern Marine Science and Engineering Guangdong Laboratory (Zhuhai), Zhuhai, 519080 China

**Keywords:** Deep ocean, Hydrothermal vents, Snail holobionts, Bacterial endosymbionts, Phages, Lysogenic and lytic infections, Anti-viral defence, Counter defence, Horizontal gene transfer, Multi-omics

## Abstract

**Background:**

Deep-sea animals in hydrothermal vents often form endosymbioses with chemosynthetic bacteria. Endosymbionts serve essential biochemical and ecological functions, but the prokaryotic viruses (phages) that determine their fate are unknown.

**Results:**

We conducted metagenomic analysis of a deep-sea vent snail. We assembled four genome bins for *Caudovirales* phages that had developed dual endosymbiosis with sulphur-oxidising bacteria (SOB) and methane-oxidising bacteria (MOB). Clustered regularly interspaced short palindromic repeat (CRISPR) spacer mapping, genome comparison, and transcriptomic profiling revealed that phages Bin1, Bin2, and Bin4 infected SOB and MOB. The observation of prophages in the snail endosymbionts and expression of the phage integrase gene suggested the presence of lysogenic infection, and the expression of phage structural protein and lysozyme genes indicated active lytic infection. Furthermore, SOB and MOB appear to employ adaptive CRISPR–Cas systems to target phage DNA. Additional expressed defence systems, such as innate restriction–modification systems and dormancy-inducing toxin–antitoxin systems, may co-function and form multiple lines for anti-viral defence. To counter host defence, phages Bin1, Bin2, and Bin3 appear to have evolved anti-restriction mechanisms and expressed methyltransferase genes that potentially counterbalance host restriction activity. In addition, the high-level expression of the auxiliary metabolic genes *narGH*, which encode nitrate reductase subunits, may promote ATP production, thereby benefiting phage DNA packaging for replication.

**Conclusions:**

This study provides new insights into phage–bacteria interplay in intracellular environments of a deep-sea vent snail.

**Video Abstract**

**Supplementary Information:**

The online version contains supplementary material available at 10.1186/s40168-021-01099-6.

## Background

Deep-sea hydrothermal vent ecosystems are abundant with macrofauna, such as invertebrate animals that adapt to such extreme environments by symbioses with chemoautotrophic bacteria [1–6]. After the discovery of hydrothermal vents in 1977, numerous studies have focused on the identification and characterisation of chemosynthetic bacteria, which have been found to have remarkable functional roles, including the ability to oxidise reduced chemical compounds to fuel the ecosystems [7–9]. Intracellular chemoautotrophic bacteria in particular have received substantial attention and have been well documented [2, 4, 10–12]. For instance, gill endosymbionts in *Bathymodiolus* mussels carry hydrogenase genes and consume hydrogen as an energy source [2]. An investigation of the mussel *Bathymodiolus azoricus* discovered two groups of endosymbionts, sulphur-oxidising and methane-oxidising bacteria, which led to dual symbiosis that gave the mussels higher environmental tolerance and the ability for niche expansion [13]. Specialised intracellular bacteria play essential roles in symbiosis with marine animals, but the bacterial viruses (phages) that determine the fate of endosymbiotic bacteria are uncharacterised.

Intracellular environments are isolated niches that protect endosymbiotic bacteria from phage infection. However, phages have been found in many endosymbiotic systems of arthropods, including flour moth [14], mosquito [15, 16], cricket [17], wasp [18], and fruit fly [19]. These phages (WO) widely infect symbiotic *Wolbachia* bacteria and enter lytic lifecycles for the parasitic A and B *Wolbachia* supergroups or lysogenic lifecycles for the C and D *Wolbachia* supergroups [20]. WO phages can be vertically transmitted or horizontally transferred between *Wolbachia* cells and possibly arthropods. WO phages have a significant impact on the abundance, activity, and diversity of *Wolbachia* and then on the symbiosis between *Wolbachia* and host arthropods [20].

The detection of potentially essential phages in the endosymbiotic systems of terrestrial arthropods suggested the possible presence of phage infection of endosymbionts that inhabit deep-sea animals. Studies on deep-sea vent fields have found snails that commonly inhabit them and develop endosymbioses with bacteria [21–25]. Snails in the genus *Gigantopelta* are abundantly colonised in Longqi [26], hydrothermal vent field at 1755 m depth in the Southwest Indian Ridge [27]. *Gigantopelta aegis* harbours two phylotypes of gammaproteobacterial endosymbionts, sulphur-oxidising (SOB) and a methane-oxidising (MOB) bacteria, in the oesophageal gland cells within the gut [24]. An omics study revealed that SOB and MOB are beneficially symbiotic to *G. aegis* snails by complementing metabolisms and providing required nutrients [24]. In the present study, we demonstrated the presence of phages that infect intracellular chemosynthetic symbionts and characterised their phylogeny and lifecycle in *G. aegis* from their genomics, transcriptomics and proteomics data. We focused on the anti-viral defence systems of bacterial endosymbionts and counter-defence mechanisms of phages to explore potential interactions between the identified phages and endosymbiotic bacteria in intracellular environments in deep-sea vent fields.

## Results

### Genomes and gene expression of phages in snail oesophageal glands

**Fig. 1:**
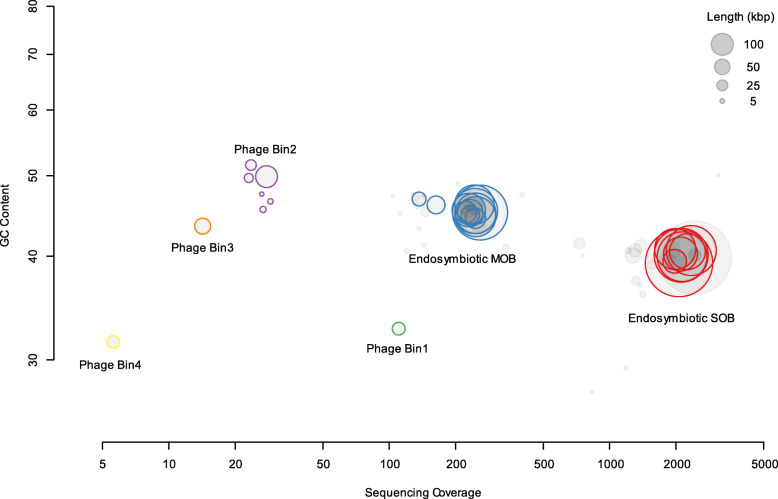
Visualisation of the four identified phage bins and endosymbiotic bacteria in a metagenomic map. One circle represents one contig sequence. Contigs of identified phages or with bacterial essential genes are marked in colour. SOB, sulphur-oxidising bacteria; MOB, methane-oxidising bacteria

Four phage bins were recovered in the present study (Fig. 1 and Table 1), and phage hallmark genes, including genes encoding portal and head proteins, were identified in each bin (listed in Tables S1–S4, visualised in Figs. S1–S5). In each bin, the genome size ranged from 29 to 139 kb, and the GC content ranged from 31% to 49%. Two of the four bins contained circular contigs, indicating that these genomes were potentially complete. The number of predicted genes for each phage bin ranged from 17 to 142. Bin2 harboured integrase-coding genes, implying that it represented a temperate phage. Transcriptomic analysis showed that phages Bin1, Bin2 and Bin4 had reconstructed transcript support (Tables S1, S2, and S4). For example, genes 1_10 for phage proteins GP46 and 1_16 encoding SNF2 in phage Bin1 were transcribed into TRINITY_DN1_c0_g1_i2 and TRINITY_DN17_c0_g1_i1, respectively (Table S1). For the temperate phage Bin2, many of its genes had transcript support, including genes for integrases, recombinases, tail proteins, baseplate proteins, lysozymes, capsids and auxiliary metabolic genes (Table S2). Although phages Bin3 and Bin4 in a relatively low abundance had less/no reconstructed transcripts detected in accordance with strict criteria (95% identity and 100% coverage), their genes showed mapping-read support according to RNA-sequencing read count (Tables S3 and S4). For instance, gene 1_12 with a length of 1173 bp for phage Bin4 capsid proteins recruited 71 reads in Sample 2 (Table S4) for mRNA sequencing.

**Table 1:** Recovered genomes of phages from gland tissues of the snail *Gigantopelta aegis*. NO, no eukaryotic or prokaryotic lineage-specific genes were detected

	Phage Bin1	Phage Bin2	Phage Bin3	Phage Bin4
Classification	*Caudovirales*	*Caudovirales*	*Caudovirales*	*Caudovirales*
Total length (Kbp)	30.130	139.757	46.730	29.326
GC content (%)	32.70	49.69	43.47	31.53
Completeness	Potentially complete (circular)	incomplete	incomplete	Potentially complete (circular)
Contamination	NO	NO	NO	NO

**Fig. 2:**
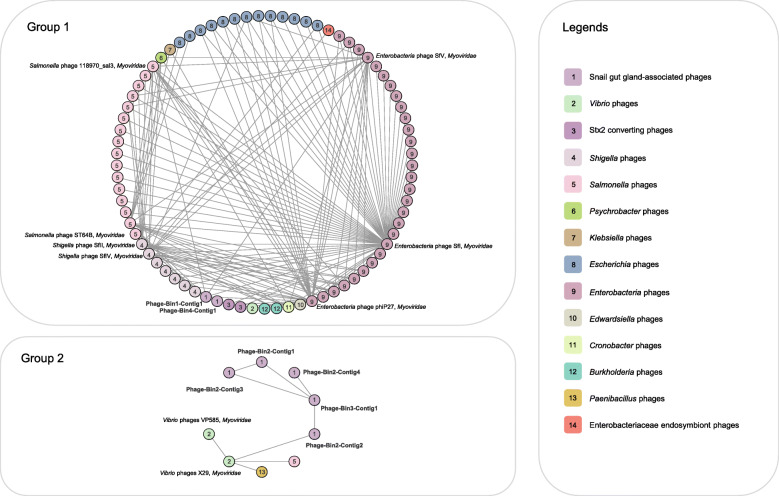
Network-based phylogeny of the four identified phage bins. Phage groups are numbered and marked in colour

Open reading frame-based taxonomic classification reveals that phage bins in deep-sea snails are affiliated with the order *Caudovirales*. Network-based phylogeny showed that these phage bins were clustered with animal/human-associated phages in family *Myoviridae* (Fig. 2 and Table S5). According to the gene-sharing network, the phages recovered in the present study were classified into two groups (Group1: phages Bin1 and Bin4; Group2: phages Bin2 and Bin3). In Group1, the snail gut-associated phages were connected directly to seven Enterobacteriaceae-infecting Myoviridae phages, namely *Enterobacteria* phages SfI, SfV, and phiP27, *Salmonella* phages 118970_sal3 and ST64B, *Shigella* phages SfII and SfIV. Notably, phages Bin1 and Bin4 were also indirectly connected to the bacteriophage APSE-2 infecting Enterobacteriaceae endosymbionts of ants, tsetse, mealybugs, and aphids. Different from Group1, Group2 included fewer members, namely, phages Bin2 and Bin3, *Vibrio* phages X29 and Vp585, *Salmonella* phage Gifsy-1, and *Paenibacillus* phage Lily. Similarly, the phages associated with phages Bin2 and Bin3 were Enterobacteriaceae-infecting *Myoviridae* phages such as *Vibrio* phage X29.

### Phage–endosymbiont connections and horizontal gene transfer

The clustered regularly interspaced short palindromic repeat (CRISPR) spacers from endosymbionts MOB and SOB were used to probe their phages by mapping to phage sequences. Phages Bin1 and Bin4 matched CRISPR spacers of both endosymbiotic MOB and SOB (Table S6). Compared with MOB, SOB contained more CRISPR spacers derived from Bin1 (12 spacers) and Bin4 (4 spacers). All the mapped phage sequences of the spacers were adjacent to the bacterial genes encoding *Cas1* and *Cas2* or *Cas6f*, which are the adaptation modules for spacer insertion [28, 29] according to the genome annotations of phages and endosymbionts (Tables S6 and S7). In addition, a phage–host link was discovered between phage Bin2 and the endosymbiont MOB because of the high similarity (3.3 kb alignment length, 100% coverage, 93% identity) between phage contig6 and MOB contig2 (Table S6).

**Fig. 3:**
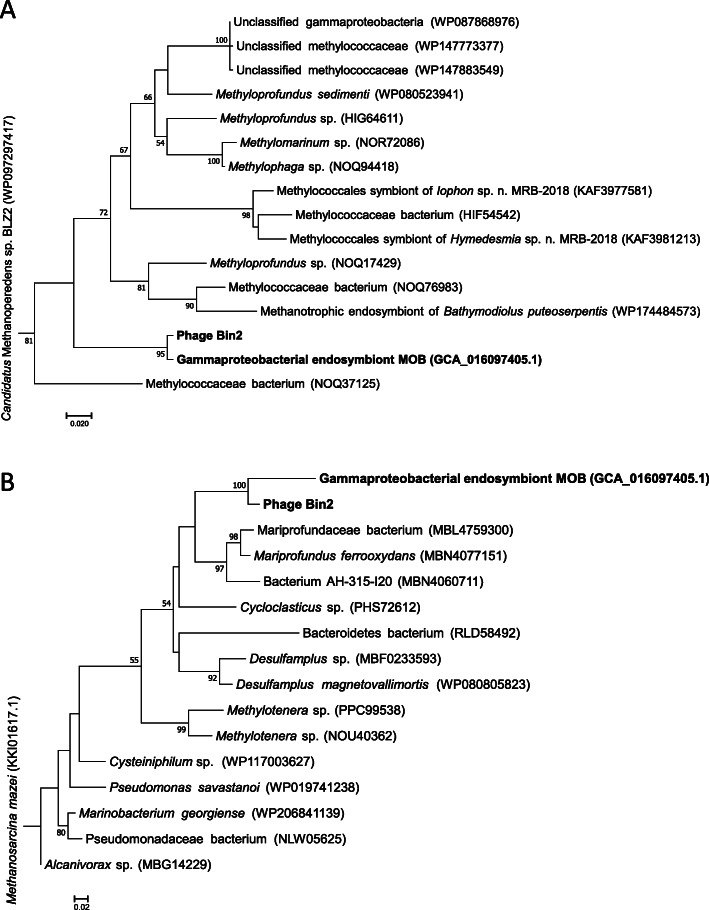
Maximum likelihood-based phylogeny of *narE* and DNA methyltransferase genes of phage Bin2 and endosymbiont MOB. **A** Phylogenetic tree of nar*H* genes. **B** Phylogenetic tree of genes encoding DNA methyltransferases. Bootstrap values <50 are not shown. MOB, methane-oxidising bacteria

Phylogenetic analysis showed that the genes for nitrate reductase subunit β (NarH) and DNA methyltransferase were horizontally transferred between MOB and phage Bin2 (Fig. 3). The *narH*, along with the gene for nitrate reductase subunit α (*narG*) located beside *narH*, is responsible for nitrate reduction in energy metabolism. The gene encoding DNA methyltransferase is related to restriction–modification systems, which are involved in innate immunity (see below). Transcriptomic analysis showed that phage *narH*, *narG*, and DNA methyltransferase genes were expressed, demonstrating that genes related to nitrate reduction and restriction-modification systems might be harnessed by phage Bin2 during the infection of MOB.

### Infection strategy of endosymbiotic phages

Thirty-eight putative prophage regions were detected in SOB and MOB using VirSorter [30], Phaster [31], and Prophage Hunter [32] (Tables S8, S9, and S10). Among them, one passed CheckV [33] examination for incomplete prophage screening based on viral-specific genes and microbial-specific genes flanked on the sides. We annotated the genes in the putative prophages against PFAM 32.0 [34] and identified phage feature genes for integrases, late control gene D protein, Gp49-like, lysozyme, tail tube protein, and tail sheath protein (Table S11). The unbinned bacterial sequences, potentially from symbionts in the metagenome, were examined by CheckV and Phaster. Two contig sequences (NODE_11432 and NODE_10) were identified as incomplete prophages. Transcriptomic analysis showed that the phage Bin2 integrase genes were expressed at relatively high levels (Table S2). Together, these results indicate that lysogeny is a potential infection strategy of endosymbiotic phages in the deep-sea vent snail *G. aegis*.

Conversely, genomic evidence for lytic infections was found. The putative prophages were compared with the phage genome bins identified in this study. However, no high genome similarity (identity ≥95%, coverage ≥80%) was found between prophages and phage bins, demonstrating that the phages and potential prophages were not from the same populations. No feature genes (e.g., integrase genes) of temperate phages were detected in phages Bin1 and Bin4, indicating they may be virulent phages with lytic lifecycles. Transcript and read mapping revealed that many genes of phages Bin1 and Bin4 for virion production had expression support, such as the genes coding portal proteins, prohead serine proteases, capsids, and head–tail joining proteins. Furthermore, the expression of lysozyme genes in phage Bin2 suggests the potential temperate phages may also have entered a lytic lifecycle and performed cell lysis to release virion progenies.

### Anti-viral defence systems in bacterial endosymbionts and their phages

**Fig. 4:**
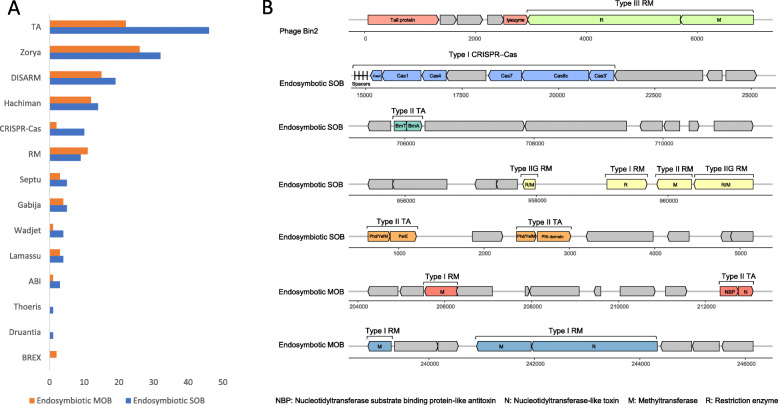
Antiviral defence systems of phage Bin2 and endosymbionts SOB and MOB. **A** Number of genes detected in each defence system in SOB and MOB. **B** Gene composition of representative sequences of defence systems with a complete set of required gene components. Non-defence genes are marked in grey. SOB, sulphur-oxidising bacteria; MOB, methane-oxidising bacteria

A total of 153 and 102 genes of anti-viral defence systems were detected in the endosymbionts SOB and MOB, respectively (Tables S7 and S12). These genes are likely involved in diverse defence systems, including Zorya, Wadjet, toxin–antitoxin (TA, Type II), Septu, restriction–modification (RM), Hachiman, Lamassu, defence island system associated with restriction–modification (DISARM), CRISPR–Cas (Type I), abortive infection, and Gabija (Fig. 4A). Among these systems, the Type II TA, RM, and CRISPR–Cas systems were complete with a full set of the required gene components (Fig. 4B). In SOB, Type II TA was composed of multiple toxin–antitoxin gene pairs for BrnT and BrnA, ParE and Phd/YefM, and PIN domain-containing proteins and Phd/YefM. In MOB, nucleotidyltransferase-like toxin and nucleotidyltransferase substrate-binding protein-like antitoxin constituted the two components of Type II TA. The foreign DNA-targeting RM systems were of two types: Type I RM (discovered in SOB) and Type IIG RM (detected in MOB). The Type I RM system encoded gene pairs for a methyltransferase that is used to modify nucleotides by methylation and a restriction enzyme for cleaving specific DNA sites. The detected Type IIG RM encoded a single gene for a combination of the methyltransferase and the restriction enzyme. The Type I CRISPR–Cas systems contained genes for Cas1–4, Cas6f, Cas7, and Cas8c. Adjacent to the genes for Cas2 (located in SOB) and Cas6f (located in MOB), an array of spacers was detected and the sequences were identical to sequences from phages Bin1 and Bin4 (Table S6). Transcriptomic analysis found that most of the defence genes (245 of 255) were expressed. For the most abundant SOB in the intracellular community, reconstructed transcript mapping and protein mass spectrometry showed that genes for the DEAD/DEAH box helicase of the Hachiman system, ZorB of the Zorya system, and Cas7 and Cas8c of the CRISPR–Cas system were transcribed and translated. Notably, the translation level of *cas7* was relatively high with an exponentially modified protein abundance index (emPAI) value of 0.93, which ranked in the top 20 of the 135 sequenced peptides of SOB and MOB in one snail sample (Table S13).

In the recovered phage bins, we also identified genes related to bacterial defence systems, with 13 genes from Bin1, Bin2, and Bin3 being involved in RM, DISARM, bacteriophage exclusion system, abortive infection, Septu, or Zorya (Table S14). The sequence alignment data were used to map 12 defence genes to meta-transcriptomic reads and nine of them had transcript mappings. Over half of the expressed genes were encoded by RM systems harboured in phages Bin2 and Bin3. Notably, the Type I and Type III RM genes for methyltransferases and restriction enzymes of phage Bin2 (Table S14 and Fig. 4B) were significantly expressed with a top 50 ranking among 142 genes (Table S2).

## Discussion

Endosymbioses are common in nature and frequently refer to beneficial, intracellular symbioses [35]. The intracellular environment is usually considered as an isolated niche that protects symbiotic bacteria from phage infection. The discovery of bacteriophages that infect endosymbiont *Wolbachia* bacteria in insects led to the study of phages in intracellular habitats of invertebrate animals [20]. Our genomic, transcriptomic, and proteomic analyses demonstrated the presence of endosymbiont phages in deep-sea snails, thereby expanding phage intracellular niches from terrestrial to marine environments.

Microbial symbionts can be horizontally transferred from the environment and/or vertically transmitted from parent hosts [36]. Accordingly, we presumed that the snail-associated phages entered the gland cells by horizontal transfer and/or vertical transmission. For horizontal transfer, the intracellular phages may be acquired by animals by uptake through the gills and guts [37], as was found in mussels, or through water flow systems, as in sponges [38]. The absorbed phages can enter animal cells, for example, through transcytosis, macropinocytosis, phagocytosis, active bacterial infection, or activation of a bacterial carrier [39]. Because symbiotic environments can select specific symbionts in horizontal transfer [36], intracellular viromes can be conserved across animal hosts, which is likely to be the reason why the snail phages are closely related to phages that infect animal-associated bacteria (Fig. 2 and Table S5). For vertical transmission, snails may acquire snail phages from their parents [20].

Previous metagenomics studies suggested the lysogenic infection strategy for phages was predominant in symbiotic systems and the model endosymbiotic WO phages were temperate phages [40, 41]. In our study of deep-sea snail endosymbiotic systems, the lysogenic lifestyles were indicated by phage integrase gene expression. We hypothesised that phage Bin2 is a temperate phage, considering the high number of transcriptomic reads identified for the integrase genes (Table S2). However, phage Bin2 did not map to any of the prophages, which suggested Bin2 may exist as a plasmid. This idea was supported by the discovery of a gene for plasmid pRiA4b ORF-3-like protein in the Bin2 genome, which was expressed with transcript support (Table S2). In Bin2, we also found relatively highly expressed genes that encoded structural proteins for virion production and lysozymes for cell lysis. Accordingly, we speculated that some of the phage Bin2 population switched back to a lytic lifecycle, whereas others maintained a lysogenic lifecycle, which is consistent with the model of spontaneous lysogenic to lytic switch [42]. Because phage Bin3 was classified along with phage Bin2 in Group2, we hypothesised that phage Bin3 is another extrachromosomal temperate phage that can be induced to enter a lytic lifecycle. However, further experimental evidence, such as plaque assays based on cultured host and virus isolates, is required to elucidate the life strategy of endosymbiotic phages in deep-sea snails.

To defend against phage infection and lysis, bacteria use their defence/immune systems as weapons to prevent population decimation by targeting invading DNA, and/or aborting viral replication, and/or conducting programmed death [43]. To target invading DNA, bacteria depend on innate and/or adaptive immunity, such as the RM [44], DND [45], DISARM [46], bacteriophage exclusion [47], and CRISPR–Cas [29] systems. To abort viral replication, bacteria have developed TA [48] and abortive infection [49] systems. Other newly found systems with unknown mechanisms have been discovered, including Zorya, Hachiman, Gabija, Septu, Thoeris, Lamassu, Druantia, Wadjet, Kiwa, and Shedu [50]. Our exploration of endosymbiont genomes showed that over 100 genes related to 14 defence systems were harboured in SOB and MOB as arsenal against phage infection and lysis. The expression profiles of the genes in these system genes suggest they co-function to form multiple complementary defence lines [51] and generate a synergistic effect to efficiently protect hosts from phage infection [43]. For example, when phages inject their DNA into SOB or MOB cells, RM systems such as Type IIG RM activate the expression of genes for restriction endonucleases to target phage DNA and cleave specific nucleic acids. Conversely, the expression of genes for methyltransferases is used to modify the host genome to avoid self-immunisation. CRISPR–Cas and RM systems are functionally coupled. The adaptive immune CRISPR–Cas system activates genes for Cas1, Cas2, and Cas7. Then, Cas1 and Cas2 incorporate partial sequences of the invading DNA of phages, such as Bin1 and Bin4, into the CRISPR array as spacers. The spacer sequences are transcribed into CRISPR coded RNA to guide Cas7 towards the invading phage DNA to cleave it. The expression of other non-DNA-targeting systems such as TA provides another line of defence. When phages successfully inject their DNA and start replication, TA systems induce the dormancy of infected cells by inhibiting gene expression; for example, the BrnT toxins in SOB inhibit protein synthesis by functioning as ribonucleases [52]. For other systems (such as Zorya and Gabija) with partially identified required gene components, the gene expression profiles indicate that they potentially have different functional roles in the phage infection. The deployment of multiple lines of defence reflects the benefits of diverse co-functioning systems when bacterial hosts are faced with diverse heterogeneous phage predators.

In the arms race of prey and predators, phages evolved counter-defence systems to resist host defences; for example, phages that carry genes of RM systems have developed anti-restriction strategies to promote bacterial infection [53]. In our study, phages Bin1, Bin2, and Bin3 contained RM-related genes for methyltransferases and restriction enzymes mostly from Type I RM systems, which were detected in their SOB and MOB hosts. The highly similar methyltransferase genes of phage Bin2 (gene 5_1) and MOB (gene 4_242; Table S15) were shown to be transferred between them (Fig. 3B), implying phages might acquire RM systems from endosymbionts. The methyltransferase-coding genes of phages were expressed at relatively high levels. These results indicate that phages may harness the expression of their genes for methyltransferases to rapidly modify the phage DNA to avoid its recognition by similar host restriction enzymes through a passive mechanism of phage evasion [53]. Moreover, the modification might protect newly synthesised phage genomes from host cleavage. The presence of RM-encoding genes may also result in a higher abundance of the phages Bin1 and Bin2 than phage Bin4, which share the same infection hosts as Bin1 and Bin2.

After successfully evading host defences, phages are faced with transcription and DNA packaging, which are energy consuming processes in the lytic replication cycle [54]. During DNA packaging, synthesised phage genomes are pumped into procapsids via ATP-dependent translocation by terminases that contain ATPase domains [54, 55]. Anaerobic respiration of prokaryotic nitrate reduction is an ATP-generating process in which the membrane-bound respiratory proteins NarG, NarH, and NarI produce a transmembrane proton motive force for ATP synthesis [56]. In the present study, Nar genes in phage Bin2 , such as *narH*, were expressed by hijacking host metabolic machinery. The Nar genes worked as auxiliary metabolic genes to generate ATP along with the highly expressed host Nar genes in the endosymbiotic MOB [24]. With the high production of ATP, the synthesised DNA and proteins can be packaged efficiently for subsequent virion assembly, which contributes to a successful lytic cycle.

## Conclusions

By genomic, transcriptomic and proteomic analysis, we assembled endosymbiotic phage genomes associated with the vent snail *G. aegis* and found that the phages had infected endosymbiotic sulphur-oxidising bacteria and methane-oxidising bacteria with lysogenic or lytic lifecycles. In the arms race between endosymbiotic bacteria and phages, the bacteria encoded CRISPR–Cas systems to target phage DNA and other potentially functional defence systems to form multiple anti-viral lines. To counter these defences, the phages evolved anti-defence mechanisms and horizontally acquired auxiliary metabolic genes to benefit replication. These findings provide new insights into phage–bacteria interplay in deep-sea vent snail holobionts. According to our understanding of phage ecology in marine environments, we expected that endosymbiotic phages might be non-animal-derived factors that regulate the population size of symbiotic bacteria in deep-sea animals.

## Methods

In this study, we reanalysed the genomic, transcriptomic, and proteomic data of the deep-sea vent snail *G. aegis*. The sampling and processing of the *G. aegis* snails have been described in detail in a previous study [24]. Briefly, the samples were collected by the Jiaolong human occupied vehicle from Longqi located on the Southwest Indian Ridge and stored at −80 °C for DNA and RNA extraction. Total DNA was extracted from the oesophageal gland containing endosymbionts using a MagAttract High-Molecular-Weight DNA Kit (QIAGEN, Hilden, Netherlands) and sequenced on an Illumina NovaSeq 6000 platform. A total of 2 × 150-bp paired-end reads was obtained. In parallel, the total DNA was sequenced using a Nanopore sequencer (Oxford Nanopore MinION, UK) for scaffolding the pre-assembled contigs from the Illumina reads. The total RNA of oesophageal gland tissues of three individuals was extracted separately using TRIzol reagent (Invitrogen, Carlsbad, CA, USA) and sequenced on the Illumina NovaSeq 6000 platform. A total of 2 × 150-bp paired-end reads was obtained. Total protein was extracted from the oesophageal glands of three individuals using methanol chloroform and analysed using a Dionex UltiMate 3000 RSLCnano and Orbitrap Fusion Lumos Mass Spectrometer (Thermo Fisher).

### Metagenome assembly and recovery of metagenome-assembled genomes of bacteriophages

The raw Illumina and Nanopore reads of the snail *G. aegis* oesophageal gland metagenome from the parent study [24] were retrieved from NCBI (BioProject accession: PRJNA612619). Trimmomatic (version 0.36) [57] was used with custom parameters (ILLUMINACLIP: TruSeq3-PE.fa:2:30:10 LEADING:3 TRAILING:3 SLIDINGWINDOW:4:15 MINLEN:40) to trim the downloaded reads. The trimmed reads were assembled using SPAdes version 3.11.1 [58] with custom parameters (--meta, k-mer size varied from 51 to 91, with a 10-mer step size). Viral sequences with hallmark genes were identified in the assembled metagenome using the VirSorter with default parameters against the RefSeqABVir and Viromes databases [30] for subsequent binning analysis. For the genome binning of phages, we followed the approach described in [59] to manually bin phage genome sequences (contigs ≥2 kb) (see processing details in Figs. S6–S9) on the basis of GC content, sequencing depth, tetranucleotide frequency, and phage hallmark genes. The binned contigs were mapped to the Illumina reads by Bowtie2 version 2.3.4 [60] and SAMtools version 1.6 [61] to select bin-related short reads. The mapped short reads along with the long Nanopore reads were used to reassemble phage bin-related sequences using Unicycler version 0.4.7 [62] with the default parameters. Sequences ≥2 kb were retained in the reassembled genomes.

In the resulting reassemblies, phage Bin2 contained more than one contig. Paired-end read information of each metagenomic contig was collected using a script (cytoscapeviz.pl with parameters: -f 2 -e 500 -m 3000 -a 125 -c) from a previous study to remove the potential contamination of eukaryotic or prokaryotic sequences in the reassembled phage Bin2 genome [59]. The eukaryotic and prokaryotic sequences in the metagenomes (after removing predicted phage contigs with hallmark genes) were identified using Autometa (parameters: --length_cutoff 1000 --maketaxtable --ML_recruitment) [63]. Two unsure contig sequences in phage Bin2 were removed because (1) the two contigs had paired-end connections to the contigs from bacterial genomes (NODE_47647 and NODE_91930), (2) they contained no phage feature genes, such as structural protein-coding genes, and (3) the sequencing coverage of the two contigs (115× and 65×) was higher than that of other contigs in phage Bin2 (approximately 25×) but relatively close to that of the connected bacterial contigs (NODE_47647 was 116×, NODE_91930 was 213×). Finally, CheckM [64] with the parameter lineage_wf was used to identify lineage-specific marker genes of prokaryotes to assess contamination from bacterial genomes. BUSCO version 4.0.beta1 with the ‘eukaryota_odb10’ database was used to check whether the viral bins lacked eukaryotic lineage-specific marker genes. The binned phage genomes were confirmed to contain no single-copy gene that was lineage specific to prokaryotes or eukaryotes. Additionally, a viral bin that contained more than one *terL* gene, indicative of a mixture of viral populations, was removed to ensure that one phage bin represented only one population. Qualified phage genome bins were retained for subsequent analysis (genome sequences were provided in Supplementary Genome sequences of Phages Bin1–4).

### Identification of prophages

Prophage regions were separately detected from the bacterial endosymbionts SOB and MOB (NCBI GenBank: GCA_016097415.1 and GCA_016097405.1) with VirSorter against the RefSeqABVir and Viromes databases, PHASTER [31]. In parallel, Prophage Hunter [32] was used to identify prophages of the endosymbiont genomes. All the identified prophage sequences were functionally annotated using HMMScan in the HMMER 3.3 tool suite [65] against PFAM 32.0 [34] (E-value <10^−3^, bit score ≥30). Prophage candidates containing phage feature sequences (e.g., attachment sites [*att*] and/or genes that code integrases and/or structural proteins) or identified as intact/active prophages were retained as prophage candidates. The candidates were further aligned with identified phage sequences using BLASTn (E-value <10^−3^, identity ≥95%, coverage ≥80%) to check whether they were from the same population to infer the lifecycle of the phages [66]. CheckV [33] was used to identify viral-specific and microbial-specific genes and ensure that the candidates were from phages integrated into host genomes. Candidates containing viral-specific genes flanked by host genes were considered reliable prophage candidates. Considering that endosymbiont sequences may not have been completely recovered from the metagenome, unbinned bacterial sequences from Autometa (parameters: --length_cutoff 1000 --maketaxtable --ML_recruitment) [63] also were examined by CheckV to detect prophage signals, to ensure a comprehensive investigation.

### Phylogenetic, taxonomic, and functional annotations of phages and bacterial endosymbionts

Following [67], we use vContact2 [68] with custom parameters (--rel-mode Diamond, --db ‘ProkaryoticViralRefSeq85-Merged’, --pcs-mode MCL, --vcs-mode ClusterONE) to cluster closely related phages. The resultant network file was imported into Cytoscape v3.8.0 [69] to visualise the network-based phylogeny of the identified phages. To analyse genome function, the genomes of the phages and endosymbiotic bacteria were imported into PRODIGAL [70] run with customised settings (-c, -m) to predict open reading frames (ORFs). The predicted ORFs were functionally annotated by searches against the Kyoto Encyclopedia of Genes and Genomes database [71] with KofamScan version 1.2.0 (E-value <10^−5^, score >predefined thresholds by KofamScan) [72]. The ORFs also were analysed with HMMScan [73] based on the PFAM database [34] with E-value <10^−3^ and bit score >30. The phage ORFs were compared with GOV-predicted proteins [74] and classified into eight categories: ‘DNA replication, recombination, repair, nucleotide metabolism’; ‘metabolism’; ‘membrane transport, membrane-associated’; ‘lysis’; ‘structural’; ‘transcription, translation, protein synthesis’; ‘other’; and ‘unknown’ [74]. To determine the taxonomy of the phages, genome bins were annotated using CAT with custom parameter settings (--fraction 0.8) [5]. Taxonomy was inferred based on searches against the NCBI-nr database (http://blast.ncbi.nlm.nih.gov/) with BLASTp (E-value <10^−5^ and identity >30%) when no taxonomic annotation was obtained using CAT.

### Transcriptome assembly, gene expression quantification, and protein identification by mass spectrometry

Sequenced RNA reads of metatranscriptome from the gland tissues of three snail *G. aegis* samples (SRR13131427, SRR13131416, and SRR13131407) from the parent study [24] were downloaded from the NCBI (https://www.ncbi.nlm.nih.gov/) Sequence Read Archive. Trimmomatic (version 0.36) was used with custom parameters (ILLUMINACLIP: TruSeq3-PE.fa:2:30:10 LEADING:3 TRAILING:3 SLIDINGWINDOW:4:15 MINLEN:40) to trim the Illumina adapters and low-quality bases of the RNA reads. Two de novo assembled transcriptomes (total-RNA-reads-assembled version and genome-bin-mapping-reads-assembled version) were independently processed with Trinity v2.8.5 [75]. For the total-RNA-reads-assembled version, all the trimmed reads were imported into Trinity with default parameters to assemble the metatranscriptomes of three samples in parallel. For the genome-bin-mapping-reads-assembled version, Bowtie2 version 2.3.4 with default parameters was used to obtain genome bin-related reads (including SOB and MOB genomes), then Trinity was used to assemble the transcriptome of each bin (phages and endosymbiotic bacteria). Reconstructed transcript sequences were mapped to the predicted ORFs of each bin using BLASTn (E-value <10^−3^, identity ≥95%, coverage =100%). Expression levels of the ORFs of the phages, SOB, and MOB were quantified in transcripts per million using Salmon [76] with default parameters and the RNA-sequencing reads as input. In this study, we defined an expressed gene as one that had the support of a transcript or read mapping. Raw mass spectrometry data of sequenced peptides were retrieved from ProteomeXchange (http://proteomecentral.proteomexchange.org/cgi/GetDataset?ID=PXD022852) from the parent study [24]. A database of predicted protein sequences from the phage, SOB, and MOB genomes with their reversed protein sequences as a decoy was constructed for searching against the sequenced peptides. The sequence database and raw mass spectrometry data were input into Mascot version 2.3.0 [77] to identify and quantify the phage, SOB, and MOB proteins (confidence ≥0.95 and false discovery rate ≤2.5%).

### Phage–host prediction

Phage–host associations between the bacterial endosymbionts and phage bins were identified using the following criteria: (1) Phage sequences from a bin and bacterial contigs from endosymbionts had ≥70% BLASTn identity (E-value ≤10^−3^) and ≥2.5 kb alignment length [74]. (2) The CRISPR spacers from an endosymbiont genome identically matched the genome sequences of a phage bin [74, 78]. MetaCRT [79, 80] was used to predict CRISPR spacers and spacers >6 bp in length were matched to phage genome bins with fuzznuc [81].

### Identification of horizontal gene transfer event

Phage and bacterial contigs were aligned using BLAST to detect horizontal gene transfer (HGT) regions. According to the criteria in [74], sequences with high alignment similarity (E-value <10^−3^, bit score >50, alignment length ≥2.5 kb and identity >70%) were retained as HGT candidates. Genes for RM-related DNA methyltransferases with a high similarity (93% amino acid identity, Table S15) between phage Bin2 (gene 5_1) and endosymbiont MOB (gene 4_242) were selected to further identify horizontally transferred genes. Putative HGT sequences were searched against the PFAM database, the Kyoto Encyclopedia of Genes and Genomes database, and the GOV classification system for functional annotation. To validate HGT events, genes for nitrate reductase subunit β (*narH*) and the DNA methyltransferases located in the putative HGT regions were used to infer a maximum likelihood phylogeny. The amino acid sequences of other *narH* and DNA methyltransferase genes were retrieved from the NCBI-nr database (http://blast.ncbi.nlm.nih.gov/) with BLASTp (E-value <10^−5^ and identity >30%) aligned with our sequences using Mafft [82] (parameters: --adjustdirectionaccurately --auto). The aligned sequences of NarH and DNA methyltransferases were then separately imported into RAxML [83] (parameters: -f a -m PROTGAMMAAUTO -N 1000) to generate maximum likelihood trees.

### Identification of prokaryotic defence system genes

BLASTp in the DIAMOND programme [84] was used to search defence system-related genes against the PADS Arsenal database [85] with custom settings (more sensitive mode, identity ≥30%, E-value <10^−10^) to investigate the diversity of the defence systems. Bacterial genes mapped to the PADS database were checked to ensure that the identified genes contained conserved domains involved in the prokaryotic defence against phages using HMMScan in the HMMER 3.3 tool suite [65] against PFAM 32.0 [34] (E-value <10^−3^, bit score ≥30). A set of PFAM accessions for the conserved domains was retrieved from a previous study [50]. Following [86, 87], putative genes for restriction enzymes and methyltransferases were detected by querying the bacterial genes against the REBASE database [88] with BLASTp (E-value <10^−6^, coverage ≥70%). The identified sequences were also searched against PFAM 32.0 using HMMScan in HMMER 3.3 tool suite (E-value <10^−3^, bit score ≥30). Sequences containing conserved domains of prokaryotic defence against phages were retained and incorporated into the set of RM-related genes. We detected the gene components of a system in a contig sequence or a bacterial bin as described previously to predict the completeness of the defence systems [89–92]. A system was considered complete if it included all the genes required for that system to function.

## Supplementary Information

**Additional file 1.** Supplementary Tables.

**Additional file 2.** Supplementary Methods and Figures.

**Additional file 3.** Supplementary Genome sequences of Phages Bin1–4. 

## Data Availability

All data generated or analysed during this study are included in this published article and its supplementary information files.

## Abbreviations

SOB: Sulphur-oxidising bacteria
MOB: Methane-oxidising bacteria
CRISPR: Clustered regularly interspaced short palindromic repeat
TA: Toxin–antitoxin system
RM: Restriction–modification system
DISARM: Defence island system associated with restriction-modification
HGT: Horizontal gene transfer
