# Supplementary material for: Arms race in a cell: genomic, transcriptomic, and proteomic insights into intracellular phage–bacteria interplay in deep-sea snail holobionts
Source: Microbiome. 2021 Sep 3;9:182. doi: 10.1186/s40168-021-01099-6 (PMC8418041; doi:10.1186/s40168-021-01099-6)
Supplement: Supplementary file 3 — Additional file 2. Supplementary Methods and Figures. [file 40168_2021_1099_MOESM3_ESM.docx]

**Supplementary Methods and Figures for:**

**Arms race in a cell: Genomic, transcriptomic and proteomic insights into intracellular phage–bacteria interplay in deep-sea snail holobionts**

Kun Zhou^1,2^, Ying Xu^2,3*^, Rui Zhang^4,5*^, Pei-Yuan Qian^1*^

^1^Department of Ocean Science and Hong Kong Branch of the Southern Marine Science and Engineering Guangdong Laboratory (Guangzhou), Hong Kong University of Science and Technology, Hong Kong, China

^2^Shenzhen University-HKUST Joint Marine Science Ph.D. Program, Shenzhen University, Shenzhen 518060, China

^3^Shenzhen Key Laboratory of Marine Bioresource and Eco-environmental Science, College of Life Sciences and Oceanography, Shenzhen University, Shenzhen 518060, China

^4^State Key Laboratory of Marine Environmental Science, College of Ocean and Earth Sciences, Xiamen University (Xiang’an), Xiamen, Fujian, China.

^5^Southern Marine Science and Engineering Guangdong Laboratory (Zhuhai), Zhuhai, 519080, PR China.

**^*^**Correspondence: boqianpy@ust.hk, ruizhang@xmu.edu.cn, boxuying@szu.edu.cn


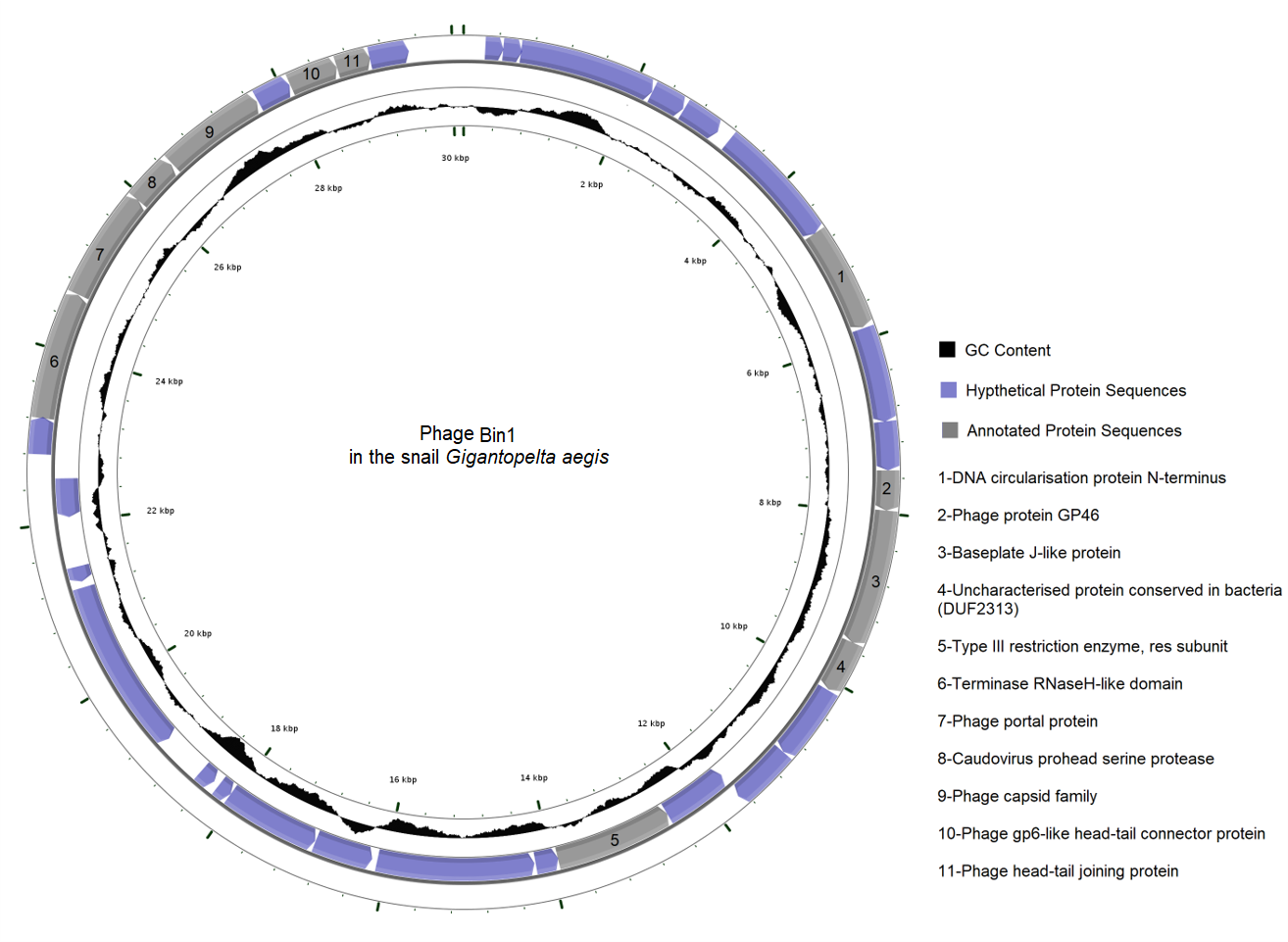


**Fig. S1 Circular genome visualization of phage Bin1.**


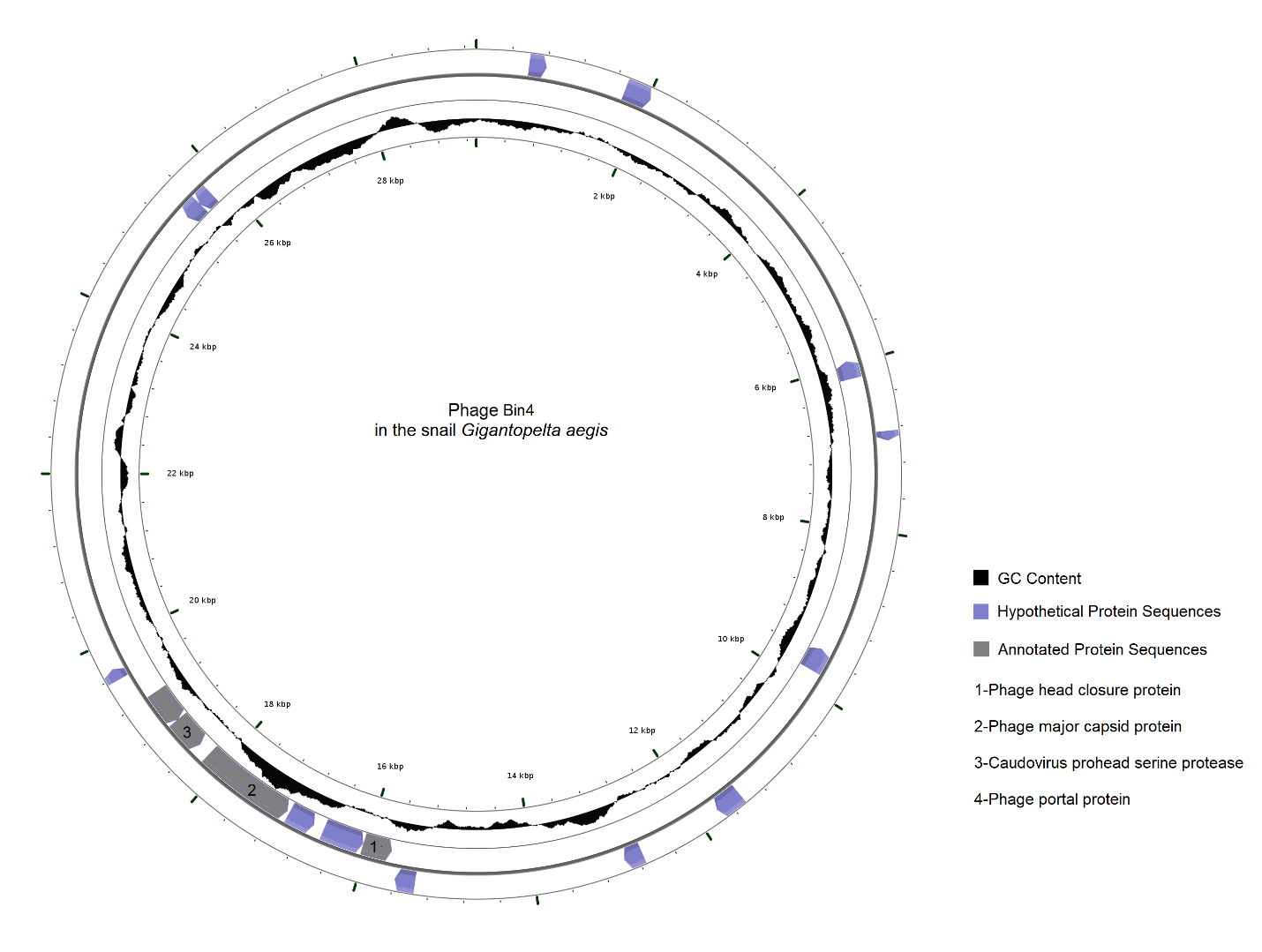


**Fig. S2 Circular genome visualization of phage Bin4.**

Contig1-1

Contig1-2

0

k

b

1

k

b

2

k

b

3

k

b

4

k

b

5

k

b

6

k

b

7

k

b

8

k

b

9

k

b

10

k

b

11

k

b

12

k

b

13

k

b

14

k

b

15

k

b

16

k

b

17

k

b

18

k

b

19

k

b

20

k

b

21

k

b

22

k

b

23

k

b

24

k

b

25

k

b

26

k

b

27

k

b

28

k

b

29

k

b

30

k

b

31

k

b

32

k

b

33

k

b

34

k

b

35

k

b

36

k

b

37

k

b

38

k

b

39

k

b

40

k

b

41

k

b

42

k

b

43

k

b

L

e

g

end:

CDS

Phage signature genes

**Fig. S3 Linear genome visualization of contig1 of phage Bin2.** As the contig1 of Bin2 is too long to be properly visualized, we broke the Contig1 into two parts (Contig1-1 and Contig1-2) for a better display.

Contig2

Contig3

Contig4

Contig5

Contig6

0

k

b

1

k

b

2

k

b

3

k

b

4

k

b

5

k

b

6

k

b

7

k

b

8

k

b

9

k

b

10

k

b

11

k

b

12

k

b

13

k

b

14

k

b

15

k

b

16

k

b

17

k

b

18

k

b

19

k

b

L

e

g

end:

CDS

Phage signature genes

**Fig. S4 Linear genome visualization of other short contigs of phage Bin2.**

Contig1

0

k

b

1

k

b

2

k

b

3

k

b

4

k

b

5

k

b

6

k

b

7

k

b

8

k

b

9

k

b

10

k

b

11

k

b

12

k

b

13

k

b

14

k

b

15

k

b

16

k

b

17

k

b

18

k

b

19

k

b

20

k

b

21

k

b

22

k

b

23

k

b

24

k

b

25

k

b

26

k

b

27

k

b

28

k

b

29

k

b

30

k

b

31

k

b

32

k

b

33

k

b

34

k

b

35

k

b

36

k

b

37

k

b

38

k

b

39

k

b

40

k

b

41

k

b

42

k

b

43

k

b

44

k

b

L

e

g

end:

CDS

Phage signature genes

**Fig. S5 Linear genome visualization of phage Bin3.**

**Binning methods for phage genomes**

Metagenomic contigs over 2000bp were selected for genome binning based on the genome differences in GC content, sequencing coverage, taxonomic assignment of prokaryotic essential genes, phage hallmark genes, and tetranucleotide frequency.

(A)
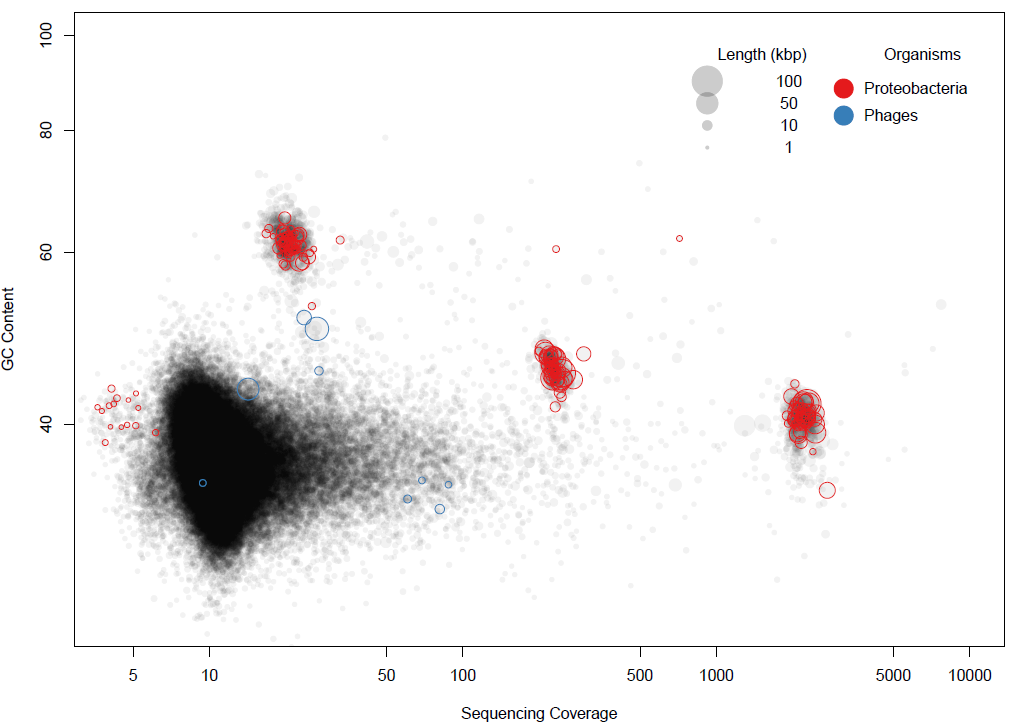
 (B)
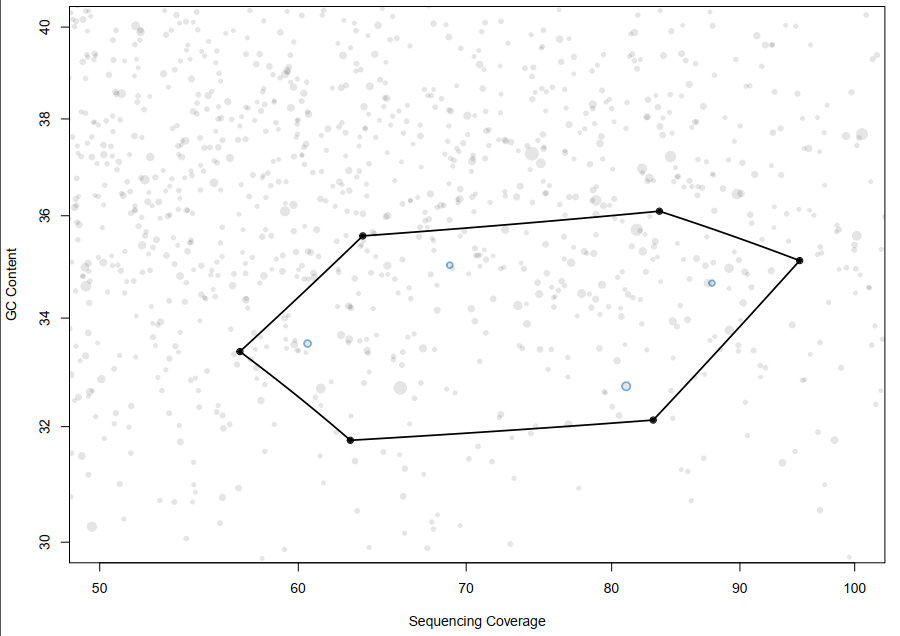


(C)
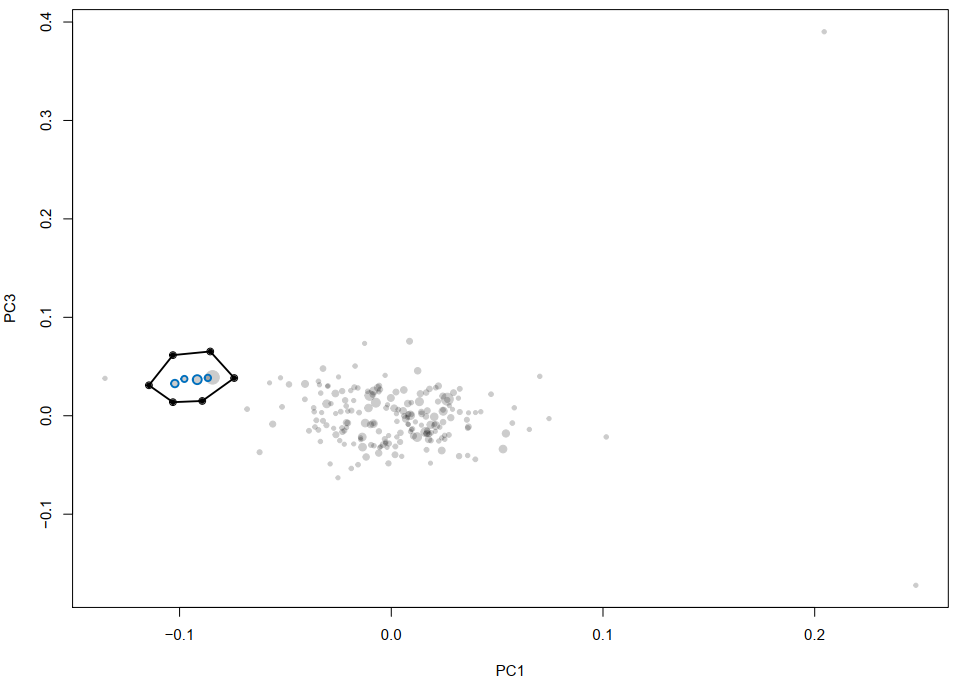
 (D)
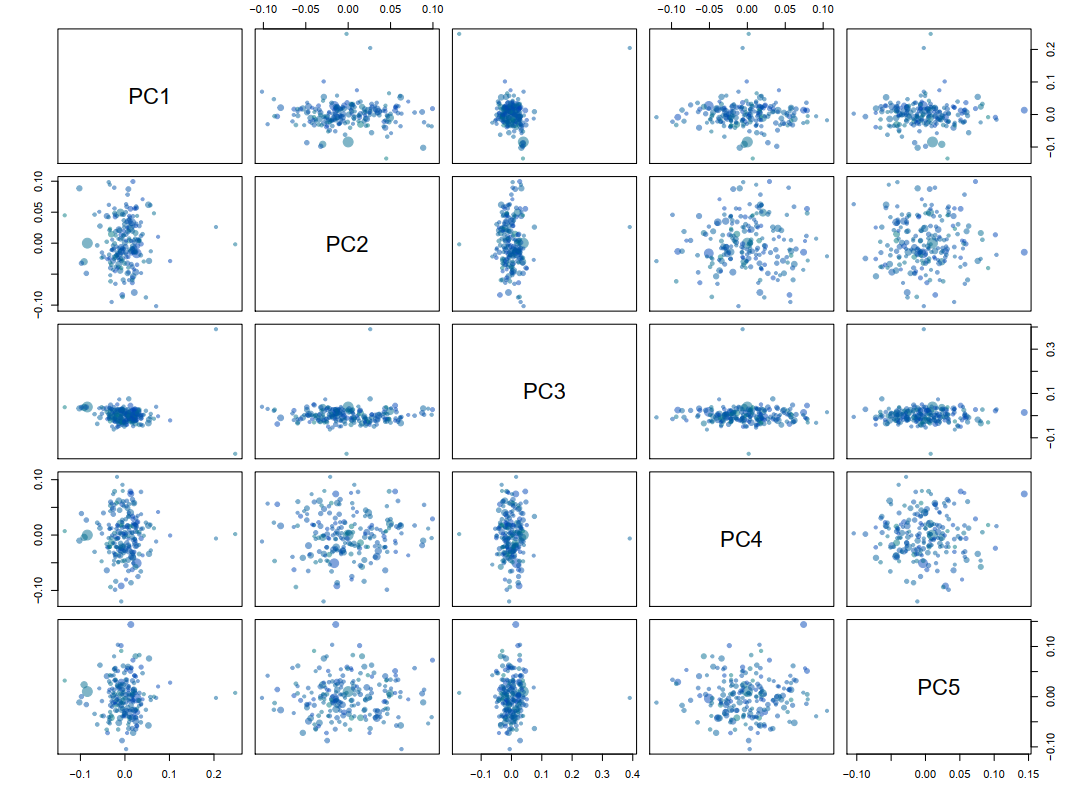


**Fig. S6 Binning for the phage Bin1.** (A) GC content and coverage plot - colored by taxonomic assignment of bacterial essential genes or phage hallmark genes. (B) Zoom in on the target genome (phage-related contigs). (C) Correspondence analysis on the subset of contigs. (D) Extract contigs using the locator. PC1 and PC3 seem to separate our target phage genome from the other contigs and are therefore used for sequence extraction using the locator function.

(A)
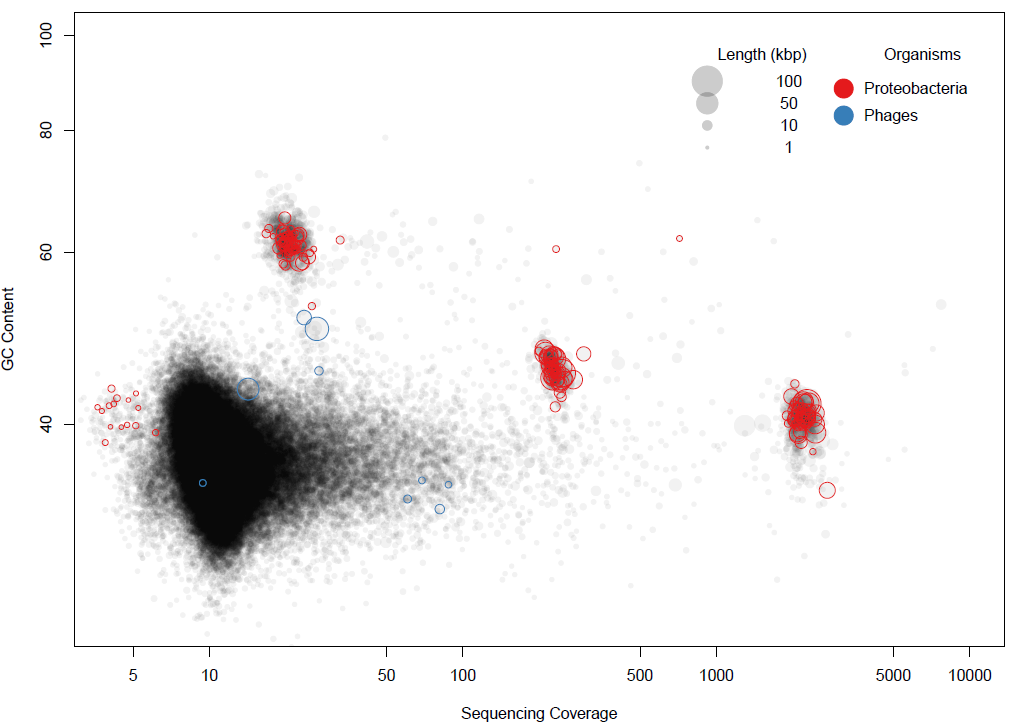
 (B)
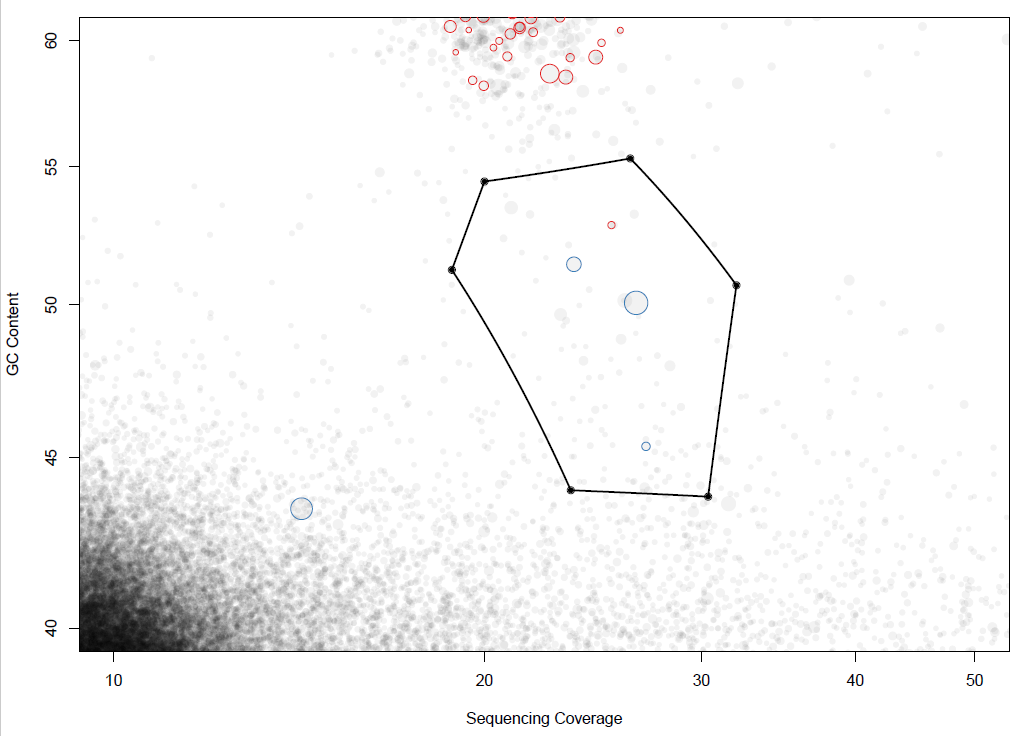


(C)
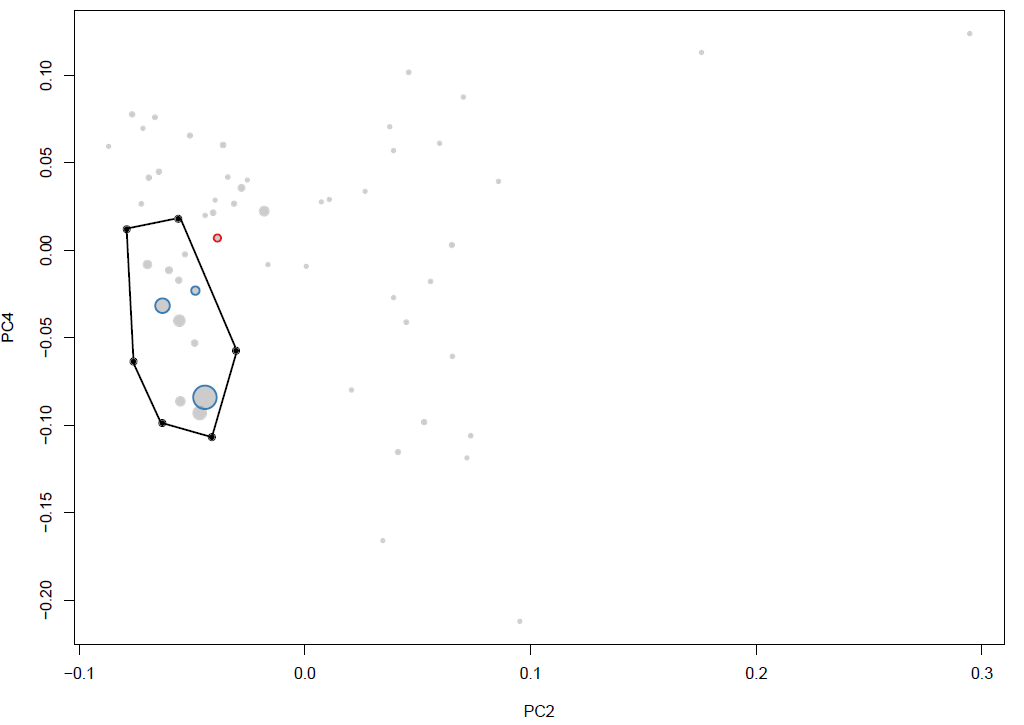
 (D)
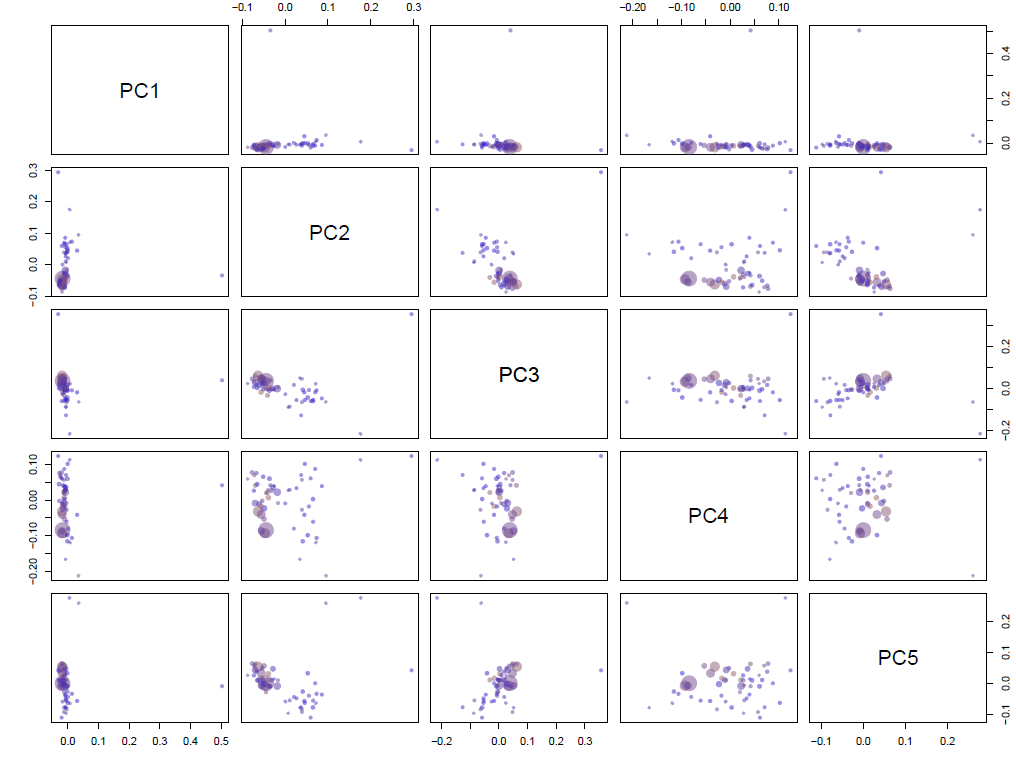


**Fig. S7 Binning for the phage Bin2.** (A) GC content and coverage plot - colored by taxonomic assignment of bacterial essential genes or phage hallmark genes. (B) Zoom in on the target genome (phage-related contigs). (C) Correspondence analysis on the subset of contigs. (D) Extract contigs using the locator. PC2 and PC4 seem to separate our target phage genome from the other contigs and are therefore used for sequence extraction using the locator function.

(A)
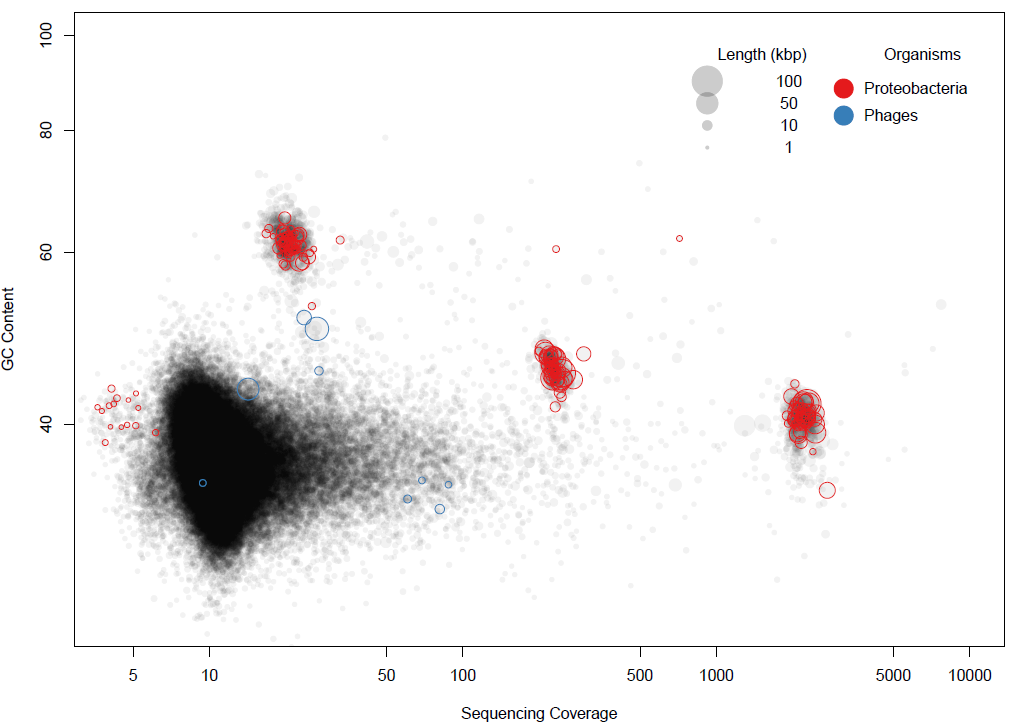
 (B)
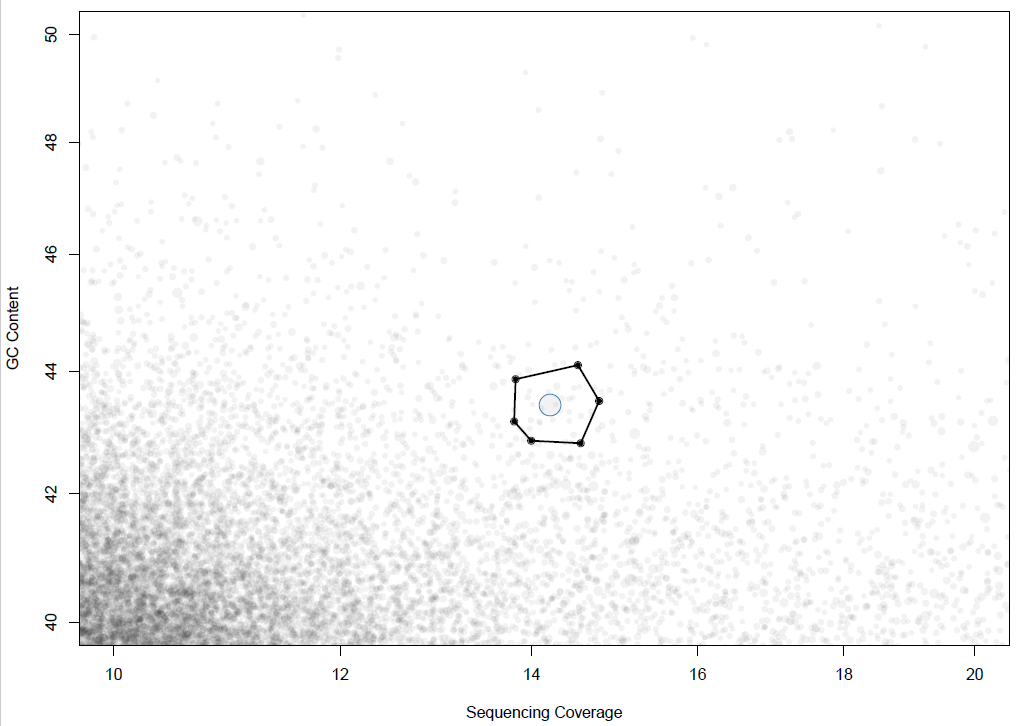


(C)
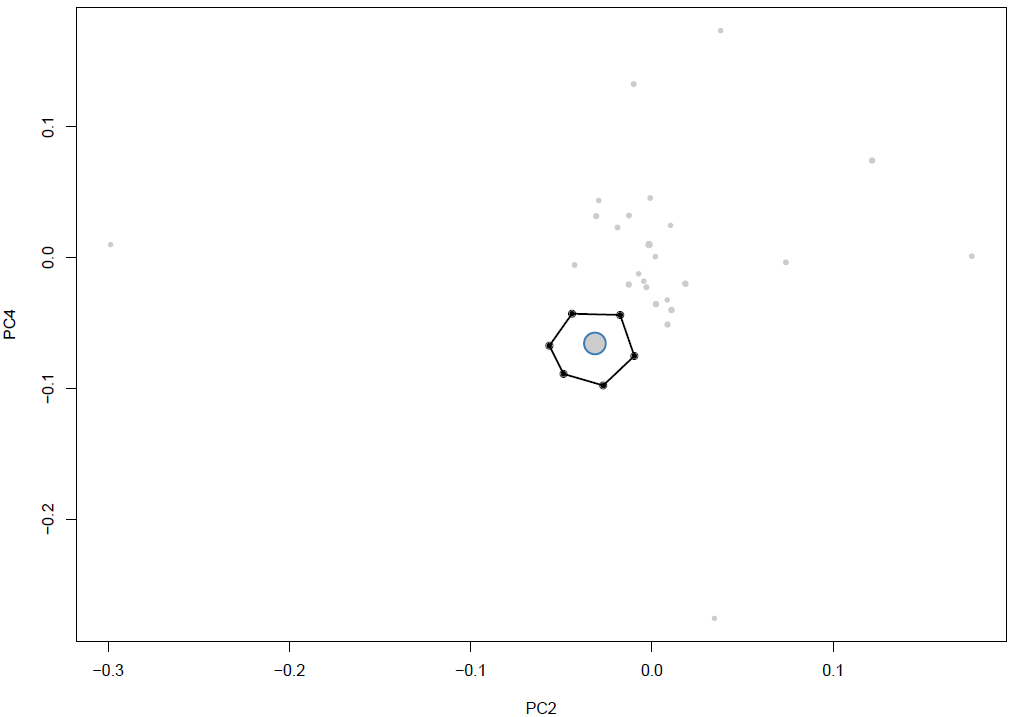
 (D)
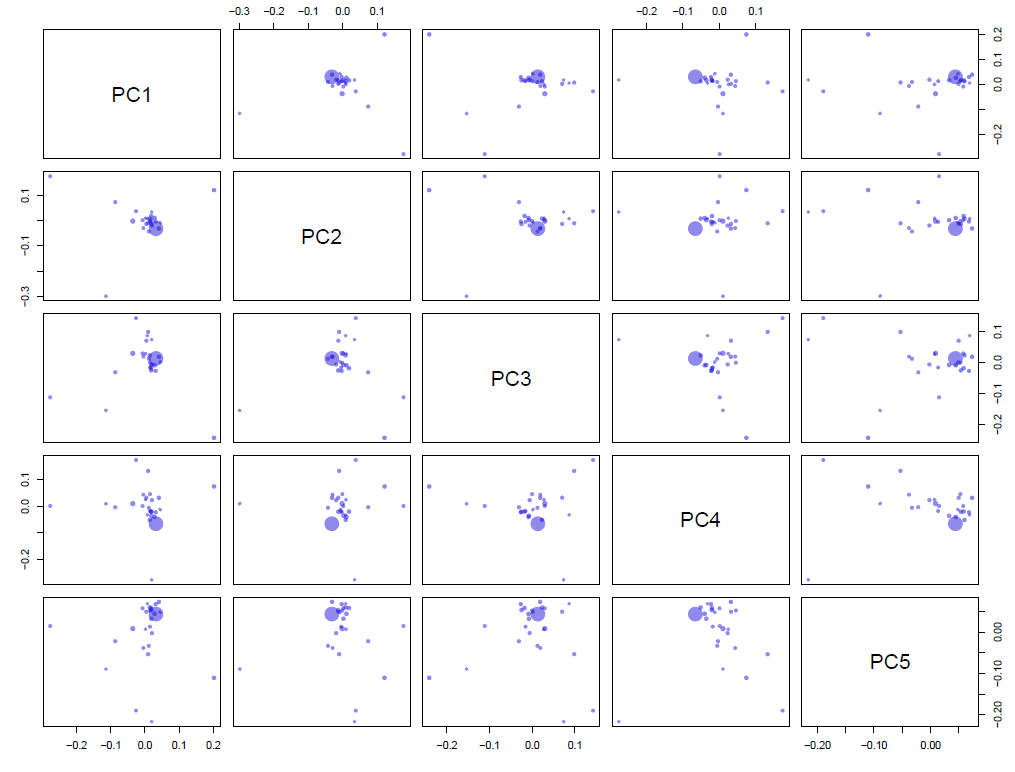


**Fig. S8 Binning for the phage Bin3.** (A) GC content and coverage plot - colored by taxonomic assignment of bacterial essential genes or phage hallmark genes. (B) Zoom in on the target genome (phage-related contigs). (C) Correspondence analysis on the subset of contigs. (D) Extract contigs using the locator. PC2 and PC4 seem to separate our target phage genome from the other contigs and are therefore used for sequence extraction using the locator function.

(A)
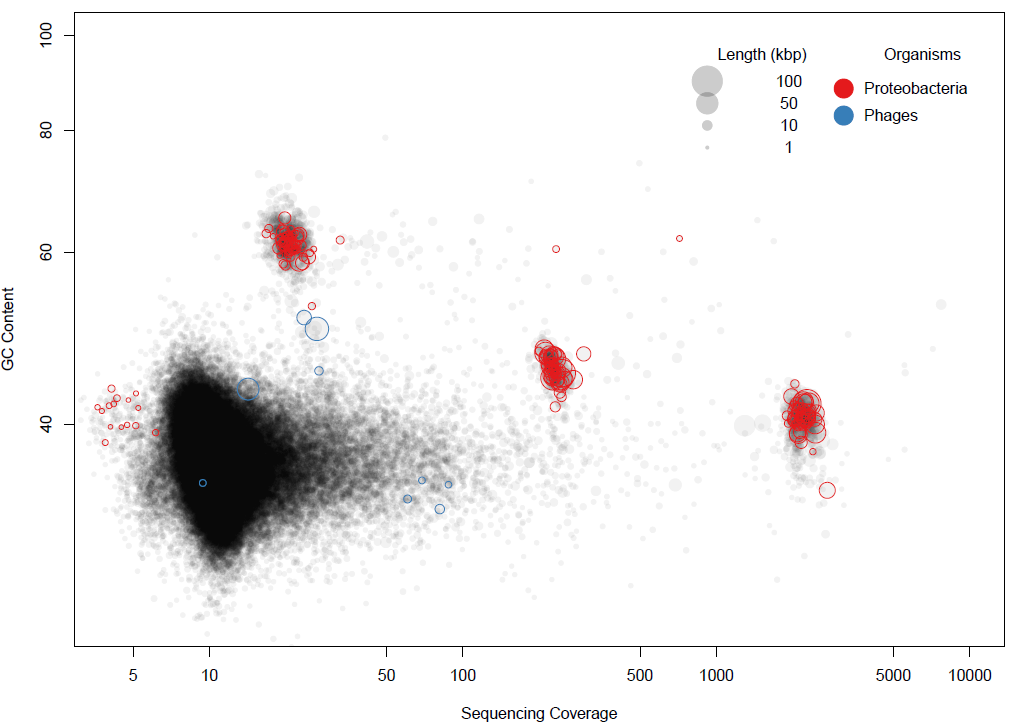
 (B)
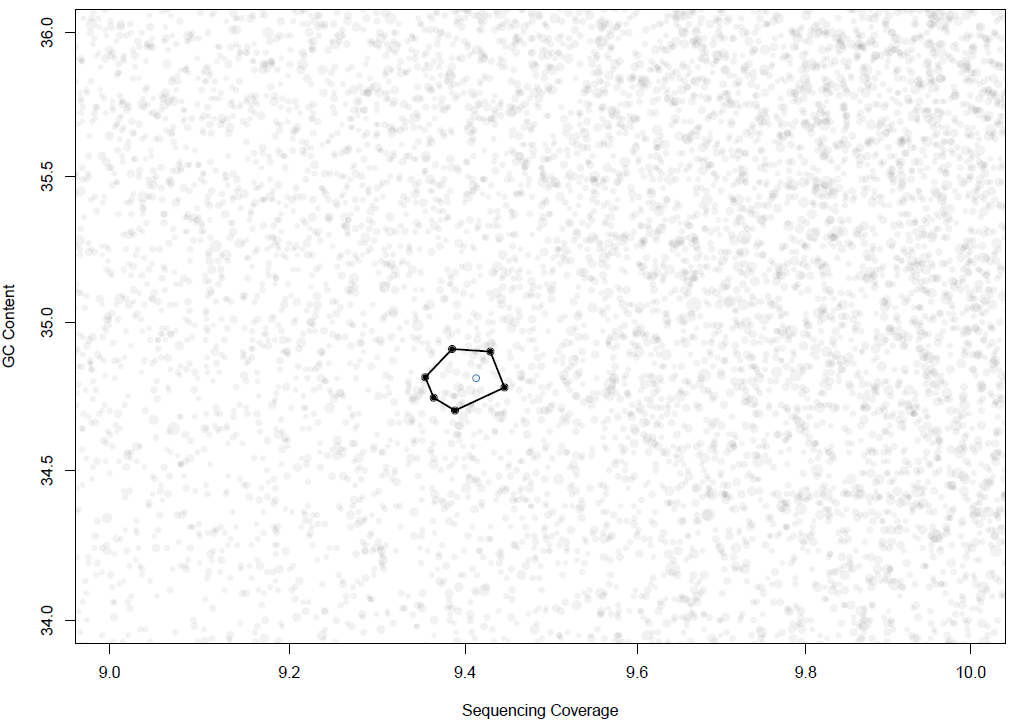


(C)
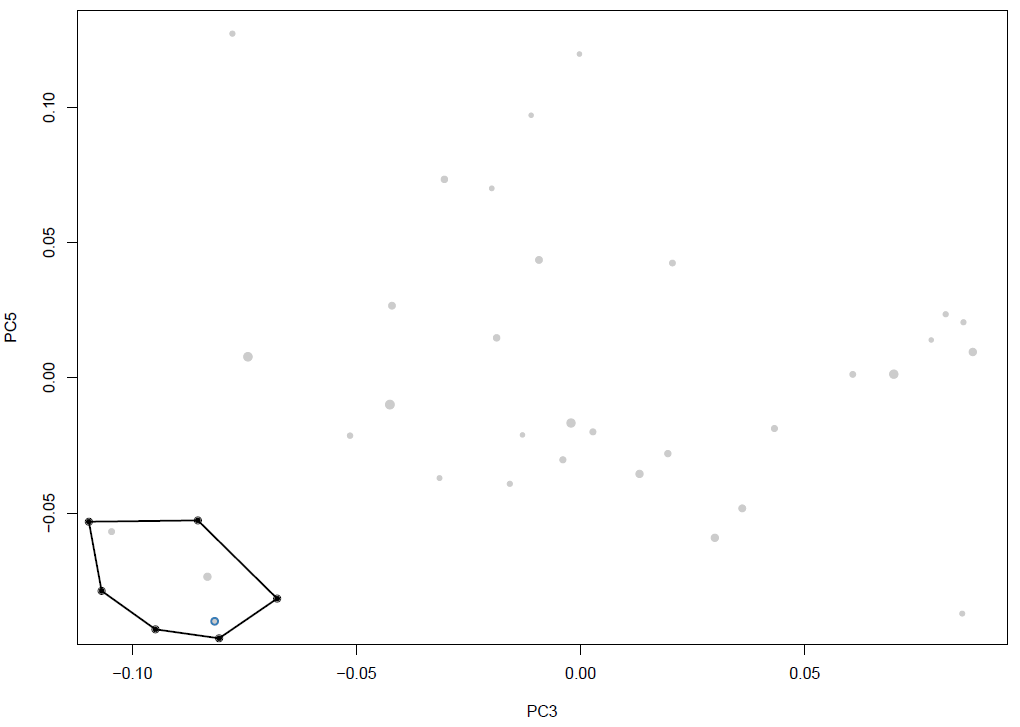
 (D)
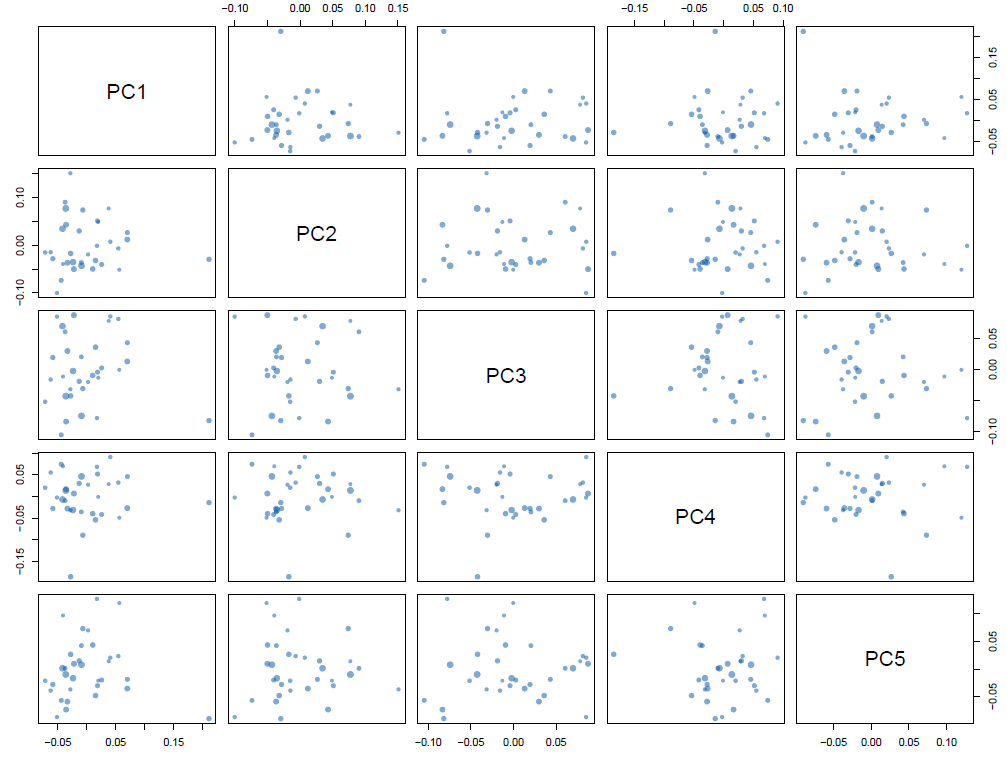


**Fig. S9 Binning for the phage Bin4.** (A) GC content and coverage plot - colored by taxonomic assignment of bacterial essential genes or phage hallmark genes. (B) Zoom in on the target genome (phage-related contigs). (C) Correspondence analysis on the subset of contigs. (D) Extract contigs using the locator. PC3 and PC5 seem to separate our target phage genome from the other contigs and are therefore used for sequence extraction using the locator function.
